# Labelling the debate: a thematic analysis of alcohol industry submissions to the EU consultation on alcohol health warnings in Ireland

**DOI:** 10.1186/s12992-025-01126-3

**Published:** 2025-05-31

**Authors:** Éadaoin Cott, Jelena Dunaiceva, Philippa White, Runa Annasdotter Neely, Matthew Lesch

**Affiliations:** 1 Department for Health, University of Bath, Claverton Down, Bath, BA2 7AY UK; 2 Department of Family Medicine, Unisanté, Center for Primary Care and Public Health, University of Lausanne, Quartier Centre, Lausanne, 1015 Switzerland; 3 Faculty of Biology and Medicine, Quartier Centre, University of Lausanne, Quartier Centre, Lausanne, 1015 Switzerland; 4 HSE Public Health, Dr Steevens’s Hospital, Steevens’s Lane, Saint James, Dublin 8, D08 W2A8 Ireland; 5 Department of Global Public Health, Karolinska Institute, Stockholm, SE-171 77 Sweden; 6 Department of Politics & International Relations, University of York, York, YO10 5DD UK

**Keywords:** Alcohol industry, Alcohol policy, Alcohol health warning labels, Industry influence, Commercial determinants of health

## Abstract

**Background:**

Building on the success of tobacco health warning labels, EU Member States and institutions are increasingly considering similar requirements for alcohol products. While industry responses to pricing and availability policies have been widely studied, their framing of Alcohol Health Warning Labels (AHWLs) as a policy solution remains comparatively underexplored. This paper examines how alcohol industry stakeholders responded to the EU notification process for Ireland’s proposed alcohol labelling regulations, introduced under Ireland’s Public Health (Alcohol) Act 2018.

**Methods:**

This paper analyses 16 submissions from alcohol industry actors to the European Commission regarding Ireland’s proposed alcohol warning label regulations. Qualitative methods, specifically thematic analysis, were used to examine industry arguments. The research team first reviewed five submissions to inductively develop a codebook, which was then applied to the remaining submissions, with new codes added as necessary. Two team members independently coded each submission, and thematic content was refined through team discussion.

**Results:**

Alcohol industry arguments against AHWLs fall into four main themes: lack of evidence supporting the content of health warning labels and their broader use, negative trade and economic impacts of AHWLs, potential risks to EU governance posed by Ireland’s labels, and the industry’s self-positioning as responsible actors committed to public health. In addition, we identify novel industry strategies related to the intricacies of AHWLs, including a heightened focus on wording and language, coordination of activities across multiple governance levels, and tailored framing to suit the institutional context.

**Conclusions:**

Alcohol industry actors employ arguments similar to those seen in other policy debates, which continue to pose a significant barrier to evidence-based alcohol policymaking. The analysis suggests that industry actors can strategically adapt their arguments to varying institutional settings and policy instruments, demonstrating their political dexterity and reinforcing the barriers to policy progress. These findings highlight the need for further research into the alcohol industry’s influence and provide insights for jurisdictions considering labelling legislation.

**Supplementary Information:**

The online version contains supplementary material available at 10.1186/s12992-025-01126-3.

## Introduction

Alcohol consumption poses a significant threat to global health. In Europe, alcohol consumption leads to approximately 800 deaths per day, and one quarter of all deaths among young adults aged 20 to 24 years are due to alcohol-attributable causes [1]. Alcohol consumption can have wide-ranging negative effects on health outcomes, directly contributing to over 200 conditions and diseases, including seven types of cancer [2]. Several evidence-based policy approaches for reducing alcohol consumption, such as regulating its affordability, marketing and availability of alcohol, are well established [3, 4]. Other interventions, however, can complement these policies. Health warning information on alcohol products can promote greater public understanding of alcohol’s health risks, empower consumers to make informed decisions about alcohol consumption [5, 6] and influence alcohol consumption behaviour [7].

Health warning labels have been shown to be highly effective policy interventions for promoting awareness of tobacco’s health harms [8–10]. This success has generated interest in alcohol health warning labels (AHWLs) in several European countries [11–15], EU institutions [16–18] and elsewhere [11, 19, 20]. Indeed, at the EU level, AHWLs have formed a key part of the regional bloc’s discussion of its 2021 Europe’s Beating Cancer Plan [21], which included a specific commitment to mandate AHWLs by the end of 2023.

At the national level, labelling policy developments have progressed the most in Ireland. In 2018, Ireland adopted the Public Health (Alcohol) Act, enacting several sweeping changes to alcohol regulation [22]. This historic public health legislation included several main provisions, including new alcohol labelling requirements, as well as the introduction of minimum unit pricing (MUP) and the structural separation of alcohol in mixed retail settings, such as supermarkets and convenience stores [12].

As an EU Member State, Ireland was obliged to seek approval for its proposed labelling regulations from the European Commission (EC) under the Single Market Transparency Directive [23]. This Directive requires EU Member States to notify all draft technical regulations concerning industrially manufactured products through the Technical Regulation Information System (TRIS) before they are adopted into national law, to prevent the creation of new technical trade barriers between Member States. Of note, Article 36 of the Treaty on the Functioning of the EU (TFEU) permits Member States to adopt a regulation for the protection of health, despite any potential trade-restricting effects, provided sound justification is given [24].

As Ireland developed its labelling regulations, the government submitted several TRIS notifications, with the final submission in June 2022. A 3-month standstill period ensued during which time any interested parties or stakeholders could comment on the proposed regulations. Despite opposition from the alcohol industry and multiple detailed opinions requesting to block Ireland’s proposed regulations, the process ended in December 2022 without any objection from the EC, thus paving the way for the implementation of AHWLs in Ireland [25]. Consequently, by May 2026 all alcohol products sold in Ireland will be legally required to carry AHWLs. These labels must include information on the health risks of alcohol consumption– specifically the risks of liver disease, fatal cancers, and drinking during pregnancy– as well as the product’s calorie content and grams of alcohol [26]. The regulations stipulate that this health information is to be provided in bold print and occupy the greatest possible proportion of the surface area reserved for both the text and symbol on the alcohol product container (see supplementary file 1 for the full labelling specifications).

Although the Irish government was successful in gaining approval from the EC for its planned AHWLs, the alcohol industry’s arguments in opposition to AHWLs during the consultative process are potentially illuminating and thus worthy of analysis. A systematic analysis can reveal how the alcohol industry frames the arguments both for and against a valuable, potentially life-saving public health intervention.

While industry opposition to population-level measures regulating pricing [27–40] and marketing [28, 41–46] has been the most studied to date, researchers have paid less attention to how alcohol industry actors have portrayed AHWLs (for an exception, see [14]). Further research is needed to explore the consistency and adaptation of the alcohol industry’s political strategies. Moreover, as alcohol labelling becomes an increasingly prominent policy issue and as other jurisdictions– including the EU– may introduce AHWLs in the future, there is a growing need for dedicated research on this topic and for enhanced scrutiny of the alcohol industry’s position on this policy. This study analyses submissions made to the EC to better understand how the alcohol industry frames AHWLs and alcohol-related issues more generally. It aims to generate a clearer understanding of how this group of key stakeholders have responded to this relatively new policy approach to alcohol control. In addition, it presents an opportunity to examine the alcohol industry’s political activities and situate these findings within the broader literature on this topic.

## Methods

To inform our approach to data collection and analysis, first we analysed other studies investigating alcohol industry submissions to global or national policymaking bodies in other contexts [27, 41, 46–51]. Second, we examined the relevant legislation and regulations, specifically Ireland’s Public Health (Alcohol) Act 2018 and Public Health (Alcohol) (Labelling) Regulations 2023. Third, we assessed the EC’s TRIS website and other relevant EC documents to develop a clearer understanding of the consultation process.

### Theoretical approach

Public health researchers have extensively studied the alcohol industry and its political activities, documenting the industry’s resistance to population-level measures regulating marketing [28, 41–46], pricing [27–40], and availability [47, 52]. These studies have helped inform broader conceptual frameworks [53, 54], including the Commercial Determinants of Health (CDoH) framework [55]. This framework explains how industry actors rely on a common set of practices– including lobbying and framing [55–61]– to advance their interests. Framing is a well-documented practice identified both within the CDoH and alcohol policy literature. Framing refers to the strategic presentation of policy-relevant information and the process by which parties develop a particular conceptualisation of or reorient their thinking about an issue. It is a strategy by which actors seek to portray specific problem-definitions and/or policy solutions in a manner that is consistent with their interests [62]. Alcohol industry actors routinely portray alcohol harm as only affecting a minority of “problem drinkers” and downplay the health harms associated with alcohol consumption [53]. Industry actors often frame population-level measures as costly regulations that unfairly penalise moderate and “responsible consumers” [30]. The effectiveness of these framing strategies has varied, with several countries, including Ireland, rejecting these arguments and instead relying on public health evidence to inform decision-making.

Thus, adopting a framing analysis approach in this study allows us to gain critical insight into how the alcohol industry presents its arguments in response to the labelling requirements proposed by Ireland’s Public Health (Alcohol) Act 2018.

### Data sources

The main data source for this study was publicly available submissions made by alcohol industry representatives to the EC in response to Ireland’s 2022 notification to the EC’s TRIS concerning Sect. 12 of its Public Health (Alcohol) Act 2018 (TRIS notification number 2022/441/IRL). These submissions were accessed via a search of the publicly accessible TRIS database [25]. The search yielded a total of 94 submissions. Of those, 89 submissions were written in English and 5 submissions in other languages, which we excluded. Several types of actors made submissions, including alcohol industry representatives, public health actors, non-governmental organisations, and individuals. Our analysis is limited to those made by the alcohol industry and written in English (*n* = 16). These submissions were downloaded as portable document formats (PDFs), saved, and imported into NVivo 14 (QSR International) for analysis [63].

### Data analysis

We used thematic analysis to analyse and compare individual submissions [64]. We followed a similar approach to O’Brien et al. (2023) in their examination of the alcohol industry’s submissions to the World Health Organization’s (WHO) Global Alcohol Action Plan consultation process [50]. Specifically, we first generated inductive codes by reviewing a subset (*n* = 5) of the 16 submissions. These codes were used to construct a codebook. Following this, two coders independently analysed 50% of the dataset (*n* = 8) each, applying the codebook or creating new codes as necessary. The results of the coding process underwent verification by three additional team members.

The classification of the alcohol industry’s argument categories was developed through an iterative, collaborative process. Initial codes were generated inductively from close readings of the consultation submissions, guided by both our research questions and existing literature on alcohol industry framing and strategies. These codes captured recurring arguments and patterns of reasoning. Through team discussions, we grouped these codes into broader categories representing distinct types of arguments. This process involved examining how individual codes related to one another and identifying underlying patterns across submissions. We then refined these categories into overarching themes and sub-themes, using a two-stage process. First, we reassessed all coded extracts in relation to the emerging themes. Second, we evaluated the themes in relation to the dataset as a whole [64]. This approach allowed us to organise and interpret the industry’s arguments while remaining attentive to both nuance and variation.

In addition to thematic analysis, we conducted descriptive quantitative analysis to capture characteristics of the submitters, including their country of origin, type of organisation, and length of submission. This allowed us to identify patterns in the data, such as the distribution of organisational types and geographic representation among submitters. Length of submission was analysed to explore the depth and complexity of the arguments presented, with longer submissions potentially indicating more detailed engagement with the topic. We also reported the frequency of key codes across submissions. This provided an indication of how common certain codes were and helped identify any discernible patterns. This quantitative approach complemented the thematic analysis, offering a more nuanced understanding of the dataset.

## Results

### Characteristics of the submitters

Submissions were primarily made by national or regional trade associations, with only one submission directly from an alcohol producer. The majority of submitters were based in the EU (*n* = 12, 75%), often headquartered in the EU capital, Brussels (*n* = 6, 37.5%). At the country level, the location of submitters was most frequently Belgium (*n* = 6, 37.5%), Ireland (*n* = 2, 12.5%), and Spain (*n* = 2, 12.5%). The median submission length was 1,424 words [IQR: 1,069–1,697 words]. Trade associations from across the sector (e.g., beer, spirits and wine industries) were all represented across the dataset (see Table 1).

**Table 1:** Overview of submissions

Submitter name	Type	Country	Length of submission (words)
Brazilian Beverage Association (ABRABE)	Trade association	Brazil	1,522
British Beer and Pub Association (BBPA) Scottish Whisky Association (SWA) Wine and Spirits Trade Association (WSTA) National Association of Cider Makers (NACM)*, **	Trade associations	United Kingdom	1,413
Comité Européen des Entreprises Vins (CEEV)	Trade association	Belgium	1,336
Conferencia Española de Consejos Reguladores Vitivinícolas (CECRV)	Trade association	Spain	1,088
Distilled Spirits Council of the United States (DISCUS)	Trade association	United States	2,592
European Federation of Origin Wines (EFOW)	Trade association	Belgium	1,010
Federación Española del Vino	Trade association	Spain	1,424
Federation of the Brewing Soft Drinks Industry	Trade association	Finland	434
Association of Alcoholic Beverage Suppliers (SAJK)*	Trade association		
FIVS***	Trade association	France	1,424
The Irish Business and Employers Confederation (IBEC) (Drinks Ireland)	Trade association	Ireland	23,985
Spirits Canada	Trade association	Canada	1,617
Spirits Europe	Trade association	Belgium	1,833
The Brewers of Europe	Trade association	Belgium	6,573
The European Cider and Fruit Wine Association (AICV)	Trade association	Belgium	647
The Shed Distillery of PJ Rigney	Producer	Ireland	890
The World Spirits Alliance (WSA)	Trade association	Belgium	1,652

*These actors made a single submission

**This submission is referred to in the Results section as “UK trade associations”

***FIVS- known formerly as Fédération Internationale des Vins et Spiritueux

### Alcohol industry arguments

Every submission (*n* = 16) from the alcohol industry expressed opposition to Ireland’s introduction of AHWLs. In analysing the submissions, we identified several overlapping arguments, which we have organised into four thematic categories.

### Theme 1. Alcohol warning labels are an ineffective, non-evidence-based policy solution to address alcohol-related problems

The evidence underpinning the content of the AHWLs is equivocal.

Industry actors claimed that warning labels are an ineffective policy measure and lack a sufficient scientific evidence base. Nearly every alcohol industry submission (*n* = 14) criticised Ireland’s proposed warning labels on the basis that these measures and the proposed information featured on them lacked a scientific basis. Specifically, industry actors argued against including information on cancer risks and alcohol consumption on labels due to a lack of evidence supporting the links between them. The Irish Business and Employers Confederation (IBEC), for example, asserted that there is no scientific consensus that “*non-excessive alcohol drinking has a direct link with cancer*.” Similarly, Spirits Canada argued against including a cancer risk warning because “*Alcohol may contribute to*,* but cannot be said to cause cancers*,* nor determine whether or not they will be fatal*.” On this point, the United Kingdom (UK) trade associations and Comité Européen des Entreprises Vins (CEEV)’s submissions used identical wording to express their concern. In their view:

*The Irish government has to date not produced any scientific evidence of a “direct link” between the unqualified consumption of alcohol and fatal cancers or causation of liver disease*,* as suggested by the wording of the proposed health warnings.*

Industry actors also argued in their submissions that the links between alcohol and disease are complex (*n* = 10) or that it is difficult to draw firm conclusions since multiple risk factors contribute to alcohol-related diseases (*n* = 9). Six of the submitters referred to cancer as a “*multi-factorial disease*.” According to CEEV, this implies that “*cancer risk cannot be evaluated in isolation*.” According to two submissions from IBEC and the Brewers of Europe, “*a single warning label*” is incapable of capturing the underlying causes of cancer risks.

Another industry objection is that Irish AHWLs fail to consider varying alcohol consumption patterns. Half of the alcohol industry submitters (*n* = 8) charged that moderate alcohol consumption does not increase disease risk. According to IBEC’s submission, for example:

*[Ireland’s proposed] warnings fail to reflect the complexities that arise in considering the health risks for consumers of alcohol*,* which vary greatly depending on the volume and pattern consumed*,* as well as the profile and background of each consumer.*

Further, Conferencia Española de Consejos Reguladores Vitivinícolas (CECRV) claimed that “*drinking wine in moderation*,* with a meal*,* as part of healthy lifestyles and dietary patterns […] does not seem to increase [consumers’] cancer risk*.”. Additionally, the Brewers of Europe’s submission took this argument a step further, suggesting that AHWLs could “*prompt reduced consumption by health-conscious moderate drinkers*,* whose consumption level may have net benefits.*”.

#### The evidence underpinning the use of ahwls is lacking

Some submissions criticised the Irish proposal for lacking sufficient evidence to warrant policy change. According to the Brewers of Europe, Ireland’s examination of the evidence on the effectiveness of AHWLs was not comprehensive enough. To support this, the Brewers of Europe referenced a single study published in the *Journal of Consumer Policy* to substantiate its claim that AHWLs are ineffective policy measures.

One-third of submitters (*n* = 6) asserted that AHWLs are weak policy interventions. According to this claim, warning labels are unlikely to achieve the intended goal of reducing alcohol consumption. Spirits Europe, for example, described health warning labels as “*ranked among the weakest [policy] interventions*” because labelling has a “*very small effect on consumers*”. The trade association representing spirit producers across the European region, however, provided no evidence to substantiate these claims.

Half of the submissions (*n* = 8) also claimed that Ireland’s AHWLs risked misleading or misinforming the public about health risks. The Distilled Spirits Council of the United States (DISCUS), for instance, criticised the proposed labels for insinuating that alcohol is the sole cause of liver disease. In their view, such claims are “*false and stigmatising.*” Similarly, the Brewers of Europe argued that the proposed labels could “*mislead consumers*” since the relationship between cancer risk and alcohol consumption is “*complex*.”

Finally, nearly three-quarters of contributors (*n* = 11) voiced concerns about the tone of the proposed warnings, arguing that because most consumers are moderate consumers, AHWLs might needlessly instil public alarm. According to DISCUS’s submission:

*Research on the efficacy of public health warnings […] shows that warning labels using alarmist language are not generally effective*,* reinforcing the need to carefully craft language that is supported by the current state of science.*

Similarly, FIVS criticised the term “*fatal*” for being “*inflammatory*” while UK trade associations described the measure as “*disproportionate*” and “*alarmist*.”

### Theme 2. AHWLs will have adverse economic impacts

Alcohol industry actors focused on the potential for AHWLs to result in negative economic impacts for different stakeholders.

#### AHWLs represent a barrier to trade

Most alcohol industry submissions (n = 11) asserted that Ireland’s proposed labelling regulations could present a trade barrier, and therefore violate existing EU law. For instance, the Brazilian Beverage Association (ABRABE) claimed that Ireland’s “*import restrictions constitute a barrier to trade and are prohibited in the EU’s single market*”. Similarly, the World Spirits Alliance (WSA) argued the measure would “*disrupt the free movement and trade flows of alcoholic beverages between Ireland and other EU Member States*”. In DISCUS’s submission, the trade association claimed that under the proposed labelling scheme, companies exporting to the EU would “*have to make special production runs*”, resulting in escalated costs. Additionally, Spirits Europe declared that “*the labelling requirements will create a barrier to trade and entry in the Irish market and have the equivalent effect to quantitative restrictions*,* which is incompatible with Article 34 and 35 of the Treaty of the Functioning of the EU (TFEU)*’’.

Many alcohol industry actors (*n* = 12) argued that the Irish government failed to consider less disruptive alternatives. For example, the Brewers of Europe argued that there are “*effective alternatives to warning labels*” that can promote public awareness of health risks while being “*less injurious to trade.*” The submission identified mass media, educational campaigns, and targeted interventions for “high-risk” alcohol consumers as examples of effective alternatives. IBEC made a similar claim, stressing the need to “*rethink the solution to the problem of alcohol consumption […] by implementing public health programmes and media campaigns*.”

Several submitters (*n* = 11) argued that the Irish government had failed to provide adequate justification for a public health exemption under Article 36 of the TFEU. For example, CECRV, the European Cider and Fruit Wine Association (AICV), and European Federation of Origin Wines (EFOW) all used identical wording in their submissions, all stating that:

*Ireland has failed to demonstrate that the measure is appropriate to protect human health and that it does not go beyond what is necessary to attain that objective. This is required should Ireland want to rely on one of the exceptions.*

#### Global economic repercussions

Industry actors’ arguments about trade implications were not restricted to the EU’s single market. Some submitters (*n* = 5) identified the negative consequences for global trade. For example, IBEC cautioned that the labelling regulation risked “*exposing Ireland and the EU to significant scrutiny at WTO level*.” This concern was echoed by WSA, which questioned the policy’s compatibility with Article 2.2 of the World Trade Organization’s (WTO) Agreement on Technical Barriers to Trade (TBT). Spirits Canada went a step further, urging Ireland and the EU to adhere to their “*international commitments*” by submitting the labelling regulation to the WTO’s TBT Committee “*for stakeholder feedback*.”

Submitters (*n* = 9) also argued that relabelling could impose prohibitive costs on alcohol producers. IBEC, for instance, claimed that imposing Irish-only labels would constitute “*an unjustified and disproportionate barrier to the free movement of goods…*” The British Beer and Pub Association (BBPA) made a similar argument, arguing that these changes would “*increase cost and complexity for both EU and non-EU producers exporting into Ireland in an already high-cost inflationary environment*.” Similarly, Spirits Europe claimed that the new labelling requirements would “*impose a disproportionate administrative and logistic burden on tens of thousands of producers of alcoholic beverages*.” Non-EU producer groups made near-identical trade-related arguments. According to ABRABE:

*The new labelling of imported products with the health warnings […] would result in the exclusion of non-Irish producers and distributors from the Irish market as this process would add costly complexity for third-country producers…*.

Several submissions (*n* = 7) warned that Ireland’s AHWLs would place disproportionate economic stress on small and medium-sized alcohol producers. For example, according to FIVS’s submission, the proposed regulations:

*… would disproportionately impact thousands of small*,* family-owned wineries*,* vineyards*,* and other businesses that would suffer increased costs and economic harm if forced to comply with the proposed changes.*

The Brewers of Europe echoed this point, explaining how the policy’s economic impacts “*would be particularly pronounced for small and medium sized economic operators*” and that this would lead to their exit from the Irish market.

### Theme 3. AHWLs create EU governance challenges

The alcohol industry made several arguments relating to how Ireland’s proposed labelling of alcohol would affect the functioning and effectiveness of EU governance.

#### Undermining EU efforts to legislate for AHWLs

All submitters (*n* = 16) argued that Ireland’s regulation could undermine current or future labelling efforts at the EU level. Several alcohol industry actors referenced the EC’s Beating Cancer Plan and associated alcohol labelling provisions. AICV stated that the EC already “*intends to propose a mandatory indication of health warnings on labels before end 2023 in the framework*” and so Ireland’s proposal “*pre-empts efforts to develop a common EU approach…*” Similarly, the UK trade associations claimed that the Irish government’s proposal risked “*superseding the intention of the Commission to legislate further in this area*.” DISCUS echoed this claim, stating that “*the Irish requirement would be duplicative of [a planned] EU-wide requirement.*”

Alcohol industry actors (*n* = 11) made a related argument that AHWLs and nutritional information should be determined centrally by the EU, namely to ensure consistency and impact of health warnings. For example, in DISCUS’s submission, it claimed that labelling regulation is best managed at “*the EU-wide level….to ensure that consumers receive consistent information*” In a similar vein, the Federation of Brewing and Soft Drinks Industry together with the Association of Alcohol Beverage Suppliers (SAJK) in Finland asserted that “*health messages and labelling […] should be applied in a harmonized manner in EU-countries*.” to avoid, inter alia, confusing consumers and reducing the impact of health messaging generally.

With both these arguments regarding the industry’s preference for harmonised regulatory standards, there also appears to be a clear effort to portray Ireland’s AHWLs as potentially encroaching upon the EU and EC’s jurisdiction.

Many industry actors also (*n* = 10) argued that creating different labelling requirements across EU countries was not in the interests of consumers. In its submission, the Shed Distillery of PJ Rigney cautioned:

*There is a risk that [our distillery] would have to [re]label [for compatibility with Irish law] only to have to be re-labelled further due to additional labelling requirements [from the EU]*,* resulting in both wasted resources and increased confusion for consumers.*

#### EU consumers should be equally informed of alcohol’s health harms

Several submissions (*n* = 6) argued that EU consumers should be equally informed about health risks. According to this argument, it would be unfair to have AHWLs in some Member States, such as Ireland, but not others. As AICV explained in its submission, consumers “d*eserve to be equally informed of any health-related issues irrespective of the Member State in which they purchase alcoholic beverages.*” Similarly, IBEC claimed that having harmonised rules for labelling is important for “*reducing inequalities in access to information for citizens across the EU*.” One Irish producer argued that “*EU consumers have a right to be meaningfully and accurately informed about the health risks of the products they consume…*”.

#### Compliance with EU law

To safeguard EU governance mechanisms and conform to existing harmonised EU legislation, industry actors (*n* = 3) firstly advocated for the incorporation of a mutual recognition clause whereby, for example, pregnancy warning labels, already mandated in other jurisdictions, would be recognised by Ireland. Spirits Europe stated, “*the Irish Bill does not include any mutual recognition principle that should apply*,* e.g. for provisions regarding the pregnancy logo on label that are already applicable in other Member States (e.g. in France and Lithuania)*.” Secondly, industry actors (*n* = 10) stipulated that if the draft regulations were to be adopted, then provision to provide a digital label should be permissible to “preserve the integrity of the single market” and thus conform to EU single market law. Spirits Europe stated:

*Digital labelling allows companies to provide detailed and more contextualised information*,* scientifically established*,* easy to access and in the language of the consumer*,* without the need to change the packaging or negatively impact movement of products within the Internal Market*.

Collectively, these industry arguments highlight the strategic positioning of their opposition to Ireland’s AHWLs as being motivated by concern for consumers, small businesses, and compatibility with EU institutional processes.

### Theme 4. The alcohol industry is a legitimate policy participant

#### Partners in public health policymaking

Alcohol industry actors (*n* = 8) commonly used submissions to position themselves as partners in public health policymaking. A key focus was portraying themselves as socially responsible actors, committed to the betterment of society. Typically, submissions began by acknowledging the issue of alcohol harm and touted the industry’s efforts to reduce this through government partnerships or financial support. In one submission, for example, UK trade associations pointed to “*numerous voluntary initiatives*” in which they work with governments and other stakeholders to reduce alcohol-related harm. Similarly, Spirits Canada outlined its financial support for different alcohol charities, including the Traffic Injury Research Foundation (TIRF) and Mothers Against Drunk Driving (MADD).

Submitters claimed broad agreement with public health aims and the need to address alcohol harm, though criticising the Irish government’s focus on population-level policy approaches. The World Spirit Alliance’s submission exemplifies this:

*[The Alliance] fully supports the collective public health objectives of encouraging adults who choose to drink to do so in moderation and responsibly*,* recognizing that some individuals should not drink at all. However*,* we question the appropriateness of the proposed warning statements…*.

Similarly, IBEC’s submission explained how it took “*no issue with the objective of raising awareness of the impact of abusive consumption of alcohol.*” Further, Spirits Canada stated that it “*supports and applauds the aim of the proposal to reduce alcohol abuse*.” In DISCUS’s submission, they acknowledged alcohol can “*result in harm*” but stressed its preferences for policy interventions that “*address and combat the harmful use of drinking*”.

Finally, some industry actors (*n* = 3) cited voluntary labelling schemes as evidence of the industry’s commitment to public health. According to IBEC:

*Many of the leading drinks producers are currently implementing labelling policies across the EU to enhance consumer transparency and awareness of the possible health risks of harmful alcohol consumption.*

## Discussion and conclusions

### Summary of findings

In this analysis of the alcohol industry’s submissions to the EU consultation on Ireland’s proposed AHWLs, we find that industry actors opposed AHWLs using several overlapping arguments that fall into four main thematic categories.

First, industry actors identified perceived weaknesses in the scientific evidence base underpinning the policy change and the effectiveness of the policy itself. The alcohol industry repeatedly disputed the association between alcohol consumption and cancer, and the health risks associated with low or moderate alcohol consumption. These claims are difficult to reconcile with existing evidence, however, there are clearly established biological pathways by which alcohol causes cancer, and a dose-response relationship has been shown between alcohol and seven different types of cancer, increasing from just one drink per day [65]. Existing research finds that alcohol causes approximately 4% of diagnosed cancers worldwide [66], and breast cancer specifically is a major contributor of alcohol-attributable cancer, causing 65.7% of female cancer cases in the European Region [65]. Additionally, the weight of existing evidence indicates that there is no level of alcohol consumption that improves health, and any purported individual benefit is offset by health risks [67, 68].

While the alcohol industry questioned the effectiveness of AHWLs as a policy intervention, there is a growing body of evidence demonstrating their effectiveness. A 2022 systematic review by Hobin et al. demonstrated that alcohol container labels with health messages, standard drink information and drink limit guidelines can improve consumer knowledge of alcohol-related risks and may influence consumers’ intentions to consume or purchase alcohol [69]. Further, a more recent systematic review by Zuckermann et al. (2024) found that AHWLs in which various health messages are frequently rotated likely substantially decrease alcohol use and alcohol sales [7]. While it is true that evidence on the effectiveness of AHWLs is still slowly emerging (progress has been stymied in part by industry interference [70]) industry actors, however, ultimately fail to justify their concerns with reference to scientific debates or, in some cases, they fail to reference any scientific literature to assert their statements. Instead, they contend that the wording of AHWLs is alarmist, substantiating these concerns by referencing two studies on communicating risk to the public, and repeatedly emphasise that the Irish government’s review of the evidence is inadequate.

Many alcohol industry actors advocated for alternative policy solutions to AHWLs, including mass media, educational campaigns and targeted interventions for high-risk alcohol consumers. Solutions of which are often coordinated by industry actors themselves [71–73]. In most instances, however, they cited a handful of studies to support these claims. Existing evidence, including systematic reviews, suggests that educational and public awareness campaigns show limited effectiveness in reducing alcohol consumption [74, 75], particularly compared to population-level policy measures, such as pricing, availability and marketing restrictions [4].

Second, submissions emphasised industry concerns regarding the potential negative economic implications resulting from the policy’s implementation. Many alcohol industry actors asserted that AHWLs would hamper the importing of alcohol into Ireland from countries in Europe and beyond, due to the cost of producing alcohol containers for the Irish market only, with a disproportionately negative economic effect on small- and medium-sized alcohol producers. Ireland’s Department of Health, however, maintains that AHWLs may feature on alcohol containers by affixing a sticker to the container after being imported into Ireland [76]. Additionally, various labelling requirements for alcoholic beverages already exist across the EU and globally, for example, health warning labels are legally required on alcoholic beverages in France and Lithuania while AHWLs have existed in the United States since the late 1980s [16, 77]. Therefore, adapting to a new labelling requirement in Ireland would not be a significant aberration from current practice. Further, alcohol producers in recent years have been agile in their ability to relabel products to align with cultural events, such as limited-edition alcohol containers for Pride Month [78] or major sporting events [79–81].

Third, the alcohol industry stressed the governance challenges that labelling could present for EU institutions and consumers. Industry actors asserted that the Irish regulations could undermine the EU’s efforts to create a common approach to AHWLs and lead to unequal understanding of the health risks of alcohol consumption across EU member states. It cautioned of impending regulatory fragmentation leading to major supply chain disruption, which would directly contravene EU Single Market Law, and claimed that Ireland did not provide sufficient grounds for the public health exemption permitted under EU law and went beyond what would be considered proportionate to achieve the objective. Given that the EU’s Beating Cancer Plan included a specific commitment to mandate AHWLs by the end of 2023 [21], any such governance issues were deemed surmountable by the EU on the basis that alcohol-related harm posed a significant threat to public health. Furthermore, as previously highlighted, AHWLs already existed in a minority of EU member states prior to Ireland’s proposed labelling regulations [82] and thus the principle of member states being able to unilaterally impose AHWLs without threatening the functioning and effectiveness of EU governance was already established.

Industry repeatedly pushed for solutions to the governance challenges they envisioned. Digital labelling and mutual recognition clauses were hailed as some of the best options for business interests and consumers alike. Research indicates, however, that information provided through Quick Response (QR) codes is not accessed by the majority of customers [83] and mutual recognition of member states’ labelling policies runs contrary to the aim of the EU’s Beating Cancer Plan seeking to harmonise rules at an EU wide level [21].

Finally, industry submissions were used to portray the alcohol industry as legitimate policy participants committed to promoting public health. Alcohol industry actors strive to position themselves as socially conscious actors. Reducing the problem to the individual level, citing “harmful”, “excessive”, “abusive” consumption of alcohol in a small number of “problem drinkers” is presented as the key issue. Health authorities have explicitly warned against using terms like “harmful use”, as they imply that some alcohol use is non-harmful. As the WHO explains:

*The contribution of alcohol consumption to cancer incidence and mortality should be clearly recognized without the use of any qualifiers or misleading adjectives such as ‘harmful’ or ‘heavy’ consumption of alcohol or ‘responsible drinking’* [84].

Evidence shows that the alcohol industry repeatedly attempts to misrepresent and distort the risks associated with the consumption of alcohol in their public communications seeking to individualise the problem and thus minimise the health harms [85] therefore relying on industry as legitimate policy participants is in direct conflict with the public health objective of reducing alcohol-related harm at a population level.

In addition to their self-portrayal as public health proponents, the industry also favours and promotes self-regulation to both enhance their credibility as actors committed to public health and to obviate the need for further public health regulation. Research finds that voluntary codes developed and adopted by industry serve to further their market preferences in lieu of public health goals [86]. Consequently, the industry’s proposed voluntary labelling schemes cannot be relied upon to provide consumers with unbiased evidence of the harms associated with the consumption of alcohol.

### Comparison with the literature

This study contributes to the existing literature on the alcohol industry and its political activities while also making several distinct contributions. We find that alcohol industry actors make arguments that are broadly consistent with previous findings [28, 41, 50, 51, 53]. As highlighted, industry actors present alcohol harm as a problem that only affects a minority of alcohol consumers with specific consumption patterns and thus portray population-level measures as misguided policy interventions. Moreover, alcohol industry actors focus attention on the economic impacts of policy interventions, with a strategic focus on smaller alcohol producers. The prevalence of these arguments in this study and elsewhere suggests a common playbook employed by the industry and worthy of further investigation [87].

Our study, however, provides several novel insights. First, by examining AHWLs, our study shows how industry actors have engaged with this relatively new policy instrument. We show how the alcohol industry’s submissions focused on the proposed label wording and raised concerns about language linking cancer risk to alcohol consumption. Our analysis also, however, reveals the industry’s relatively superficial engagement with the scientific evidence on this topic. Rather than challenging the health-harming consequences of alcohol consumption, alcohol industry actors emphasise the “complexity” of disease risks, thereby arguing against the necessity of population-level measures such as AHWLs. This is broadly consistent with other studies on industry framing techniques [28, 41, 88] but ought to be more carefully investigated in the context of labelling policy.

Second, our study suggests that alcohol industry actors operate at multiple levels of governance to actively coordinate their arguments. Our analysis of a small number of submissions allowed us to identify key similarities across submissions. In several cases, submissions contained identical wording and/or formatting. Previous research has identified trade associations and Social Aspect Public Relations Organizations (SAPROs), including the International Alliance for Responsible Drinking (IARD), as key coordinating bodies linking disparate groups [89, 90]. Our findings underscore the need for additional research on the extent to which these actors rely on identical arguments and the mechanisms enabling these actors to coordinate their political strategies.

Third, our analysis provides insight into how the institutional context often shapes the alcohol industry’s framing tactics. In our case study, industry actors claimed that Ireland’s approach to labelling could threaten EU-wide approaches to labelling. The EC’s remit to prevent trade barriers meant that the alcohol industry had clear incentives to portray alcohol labelling by employing economic or trade-related frames. According to submissions, the alcohol industry supports the EC’s proposal to proceed with mandating the inclusion of health warning labels. On the surface, this might be surprising, given the submitters’ concern about the effectiveness of labelling described above. On the other hand, it likely reflects the industry’s strategic considerations. Discussions about labelling requirements are still in the early stages at the EU level. EU policies require the reconciliation of several competing viewpoints, including major alcohol-producing countries. Industry actors, then, might see this debate as an opportunity to shape and/or weaken labelling requirements. Existing research in public policy, specifically on venue-shopping, is particularly instructive here. According to Punctuated Equilibrium Theory, policy actors opposed to a policy change often seek out alternative policymaking venues that may be more sympathetic to their preferred framings or policy solutions [91, 92]. Our research demonstrates how alcohol industry actors criticised Ireland’s AHWLs on procedural grounds and sought to move the decision-making to a venue that they believed might be more favourable to their interests as it promotes stakeholder consultation in the development of EU regulations [93].

It is also noteworthy that these alcohol industry activities closely resemble strategies undertaken, for example, historically by the tobacco industry to stymie the introduction of health warnings on tobacco packaging [94–96] and by the food industry in response to front-of-pack nutritional labelling (FOPNL) [97–99]. Reassuringly, many countries have surmounted this corporate influence and have proceeded to implement health warnings on unhealthy commodity products [100–102].

### Public health implications

Our findings have implications for the public health research community generally. Evidence shows that corporations, including the alcohol industry, have promoted the use of good governance type mechanisms such as the EU ‘Better Regulation’ agenda which mandates stakeholder consultation, impact assessments and promotes the reliance on scientific evidence to develop regulations specifically in order to create difficulties for governments attempting to pass public health and environmental policies [103, 104]. While in essence these processes should facilitate transparent and evidence-based policy development, in reality they offer industry a voice in the development of public health regulations and a platform to disseminate frequently misleading evidence, which collectively serve to further their market-driven objectives over population health. Furthermore, the requirement to provide impact assessments, focusing on the economic costs of a potential policy, favours industry interests ahead of public and environmental health [94, 95, 105]. Several mechanisms can work to minimise this corporate effect on the development of public health policies. These include strategically mobilising civil society groups to strengthen the public health advocacy in consultations, requiring mandatory conflict of interest declarations with each submission, imposing transparency rules for submitters to disclose their funding sources, and appointing designated researchers within public health teams to coordinate responses in close consultation with civil society organisations [106].

The scientific evidence on labelling’s effectiveness is not as well established when compared to pricing and availability. Viewed this way, industry actors might see this limited evidence as a potential vulnerability or opportunity (akin to former and continued efforts by the tobacco industry to avert tobacco control policies [107, 108]. One potential consequence is increased industry involvement in funding scientific research on the effectiveness of labelling. The alcohol industry has a long history of efforts to shape the evidence base on alcohol consumption and policy [53, 109–111]. It’s clear from our study that the alcohol industry is relying on these decision-making processes to shape and stymie alcohol policy progress. Therefore, public health researchers need to be particularly attentive to these dynamics related to AHWLs and public health policies more generally.

We have demonstrated that the alcohol industry often exploits gaps in the existing literature to downplay the connection between alcohol consumption and cancer risks. For instance, the industry highlights research gaps concerning cancer risks among individuals who lead otherwise healthy lifestyles, suggesting that regular physical activity and balanced diets might mitigate the cancer risks associated with alcohol consumption. More nuanced research in different population groups is needed to understand how factors such as lifestyle, genetics, socio-economic status, and pre-existing health conditions interact with alcohol consumption to influence cancer risk. Such detailed studies will both expand the evidence base and strengthen effective response to the alcohol industry’s arguments and therefore facilitate the implementation of labelling in other countries. Nevertheless, a lack of such evidence should not preclude EU member states from developing alcohol labelling regulations, as the precautionary principle provides for such an instance [112].

### Limitations

Several limitations of this study should be borne in mind. First, our study only provides insight into the claims and arguments made in these submissions but does not examine other interactions and engagements that industry actors may have had with EU officials, especially given an established industry lobby at this level [113]. The absence of submissions from alcohol retailers is striking. It is possible that some industry actors, particularly retailers, have used other avenues to shape decision-making at the EU level. Second, our analysis does not systematically study the degree to which industry submissions are similar. Future research could use topic modelling, corpus linguistics, or similar methods to provide fine-grained analyses of the submissions, examining how wording and other patterns characterise industry submissions.

## Conclusion

Labelling appears to be the next major policy battle between the alcohol industry and public health actors. Researchers, practitioners and the general public require a clearer understanding of the arguments and tactics employed by the alcohol industry to better counter these strategies and support the reduction of alcohol-related harm in our societies.

## Electronic supplementary material

Below is the link to the electronic supplementary material.

Supplementary Material 1

## Data Availability

All data generated or analysed during this study are included in this published article or in the hyperlinks provided.

## Abbreviations

AHWLs: Alcohol Health Warning Labels
MUP: Minimum Unit Pricing
EC: European Commission
TRIS: Technical Regulation Information System
TFEU: Treaty on the Functioning of the European Union
CDoH: Commercial Determinants of Health
PDFs: Portable Document Formats
WHO: World Health Organization
WTO: World Trade Organization
TBT: Technical Barriers to Trade
ABRABE: Brazilian Beverage Association
BBPA: British Beer and Pub Association
SWA: Scottish Whiskey Association
WSTA: Wine and Spirit Trade Association
NACM: National Association of Cider Makers
CEEV: Comité Européen des Entreprises Vins
CECRV: Conferencia Española de Consejos Reguladores Vitivinícolas
DISCUS: Distilled Spirits Council of the United States
EFOW: European Federation of Origin Wines
SAJK: Association of Alcoholic Beverage Suppliers
FIVS: Fédération Internationale des Vins et Spiritueux
AICV: The European Cider and Fruit Wine Association
WSA: World Spirits Alliance
IBEC: The Irish Business and Employers Confederation
TIRF: Traffic Injury Research Foundation
MADD: Mothers Against Drunk Driving
QR: Quick Response
SAPROs: Social Aspect Public Relations Organizations
IARD: International Alliance for Responsible Drinking
FOPNL: Front-of-Pack Nutrition Labelling
