# Supplementary material for: Labelling the debate: a thematic analysis of alcohol industry submissions to the EU consultation on alcohol health warnings in Ireland
Source: Global Health. 2025 May 31;21:34. doi: 10.1186/s12992-025-01126-3 (PMC12125837; doi:10.1186/s12992-025-01126-3)
Supplement: Supplementary file 1 — Supplementary Material 1 [file 12992_2025_1126_MOESM1_ESM.docx]

**S.I. No. 249/2023 - Public Health (Alcohol) (Labelling) Regulations 2023**

Conditions for label on alcohol product containers:


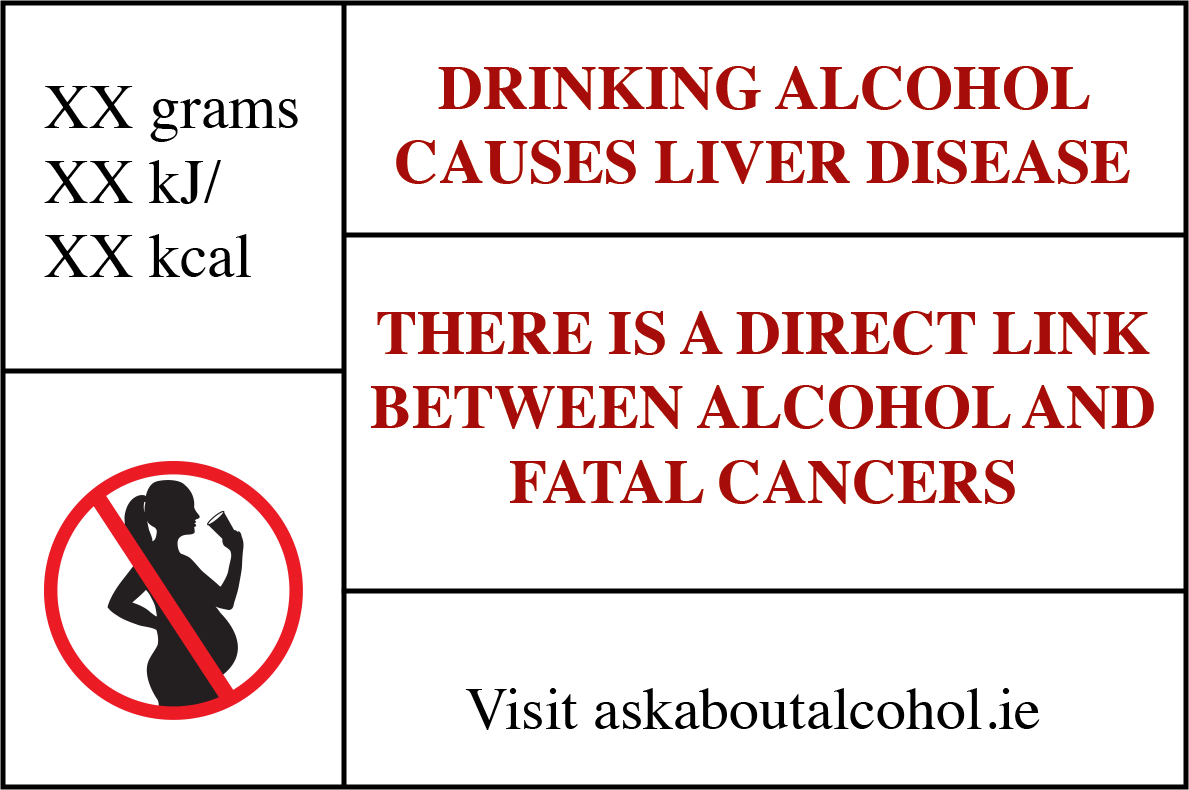


The Government of Ireland has specified that each component of the health warning label on alcohol product containers follow the requirements outlined in box 1. The combined surface area for the health warnings, information and symbol is set to a minimum of 60 mm in width and minimum 30 mm in height, to be surrounded by a black border. The label can be included either as part of the manufacturer’s label secured to the alcohol product, included on a sticker affixed to the container of alcohol or marked/stencilled/embossed or impressed to the container.

| Component of Health Warning | Predominant Requirements |
| --- | --- |
| Drinking Alcohol Causes Liver Disease | Printed in Times New Roman bold type on white background  Printed in upper-case  Printed in colour pantone reference 2035 or red like colour  Printed in largest font size to occupy the greatest possible area reserved for text  Positioned at the centre of reserved space and in same direction as other information.  Have a width of no less than 45mm and height of no less than 10mm and 15mm respectively  Shall not contain anything other than symbol |
| There is a direct link between alcohol and fatal cancers |  |
| Symbol | Printed in black & pantone reference 2035 or red like colour  Printed on white background  Printed so that the red line line crossing the human figure does not obscure the figure.  Positioned at the centre of the reserved space and in the same direction as text.  Have a width of no less than 15mm and height of no less than 15mm  Shall not contain anything other than symbol |
| Quantity of Alcohol in Container | Printed in black Times New Roman on white background  Printed in largest font size to occupy the greatest possible area reserved for text  Positioned at the centre of reserved space and in same direction as text.  Have a width of no less than 15mm and height of no less than 15mm  Shall not contain anything other than specified information |
| Relevant Website | Printed in black Times New Roman on white background  Printed in largest font size to occupy the greatest possible area reserved for text  Positioned at the centre of reserved space and in same direction as text.  Have a width of no less than 45mm and height of no less than 5mm  Shall not contain anything other than specified information |

Reference: Government of Ireland, 2023.  Public Health (Alcohol) (Labelling) Regulations, (2023). [Available from: <https://www.irishstatutebook.ie/eli/2023/si/249/made/en/print>
